# Multimorbidity patterns and health care utilization among older adults with schizophrenia

**DOI:** 10.1192/j.eurpsy.2023.811

**Published:** 2023-07-19

**Authors:** A. Hwong, Y. Li, R. Morin, A. Byers

**Affiliations:** 1 Psychiatry and Behavioral Sciences, University of California, San Francisco; 2 San Francisco VA Medical Center; 3 Northern California Institute for Research and Education, San Francisco; 4 Hoag Hospital, Irvine, United States

## Abstract

**Introduction:**

Older adults with schizophrenia often have multiple chronic conditions, or multimorbidity, yet most prior research has focused on single medical conditions.

**Objectives:**

To characterize multimorbidity patterns and utilization among older adults with schizophrenia to understand how multimorbidity affects this population and their clinical service needs.

**Methods:**

This retrospective cohort study included veterans aged 50 years and older with schizophrenia and followed their comorbid diagnoses and utilization (outpatient, inpatient, and emergency) from 2012 to 2019. Comorbid diagnoses included myocardial infarction, congestive heart failure, stroke, chronic obstructive pulmonary disease (COPD), cancer, dementia, traumatic brain injury, hepatitis C, osteoarthritis, renal disease, chronic pain, sleep disorder, depression, dysthymia, posttraumatic stress disorder (PTSD), general anxiety disorder, alcohol use disorder, other substance use disorder, and tobacco use disorder. Latent class analysis was used to identify latent profiles of psychiatric and medical comorbidity. Chi-square and F-tests were used to assess differences in demographics, comorbidities, and utilization across the latent classes.

**Results:**

The cohort included 82,495 adults with schizophrenia. Three distinct multimorbidity classes were identified: Minimal Comorbidity (67.0% of the cohort), High Comorbidity (17.6%) and Substance Use Disorders and Related Conditions (SUDRC) (15.4%). The Minimal Comorbidity class had <10% prevalence of all comorbid diagnoses. The High Comorbidity class had >20% prevalence of congestive heart failure, COPD, dementia, renal disease, sleep disorder, and depression. The SUDRC class had >70% prevalence of alcohol and drug use disorders and >20% prevalence of COPD, hepatitis C, depression, and PTSD. Although the High Comorbidity class had the highest rates of chronic medical conditions, the SUDRC class had the highest rates of emergency and inpatient medical care and emergency, inpatient, and outpatient mental health care utilization. Comparing across classes, all p-values were <.001 for utilization.

**Conclusions:**

Older adults with schizophrenia are a heterogeneous group with distinct multimorbidity classes and different patterns of utilization. Those with high prevalence of substance use disorders had the highest rates of emergency and inpatient medical and overall mental health care utilization. Tailoring integrated care services to target specific clinical needs could improve outcomes for this population.

**Disclosure of Interest:**

None Declared

